# Синдром Донохью. Описание клинического случая и опыт применения непрерывной подкожной помповой терапии рекомбинантным ИФР-1

**DOI:** 10.14341/probl13121

**Published:** 2022-06-22

**Authors:** М. А. Меликян, Т. Е. Иванникова, Н. В. Милованова, А. A. Колодкина, О. Б. Безлепкина, Н. Г. Мокрышева

**Affiliations:** Национальный медицинский исследовательский центр эндокринологии; Национальный медицинский исследовательский центр эндокринологии; Медико-генетический научный центр имени академика Н.П. Бочкова; Национальный медицинский исследовательский центр эндокринологии; Национальный медицинский исследовательский центр эндокринологии; Национальный медицинский исследовательский центр эндокринологии

**Keywords:** синдром Донохью, инсулинорезистентность, инсулиновый рецептор, рекомбинантный ИФР-1

## Abstract

Синдром Донохью (лепречаунизм) — это наиболее тяжелая форма инсулинорезистентности, возникающая вследствие мутаций в гене рецептора инсулина INSR (OMIM: 147670). Данное заболевание встречается с частотой 1:1 000 000 живых новорожденных. У пациентов отмечается типичная клиническая картина: выраженная задержка внутриутробного развития, дизморфизм, грубые метаболические нарушения, гепатомегалия, гипертрофия миокарда. Большинство пациентов погибают в течение первых двух лет жизни вследствие интеркуррентных инфекций верхних дыхательных путей, эпизодов тяжелых гипогликемий или кардиомиопатии. В настоящее время не существует специфической терапии для лечения синдрома Донохью, однако в мировой литературе имеется ряд описаний успешного применения рекомбинантного инсулиноподобного фактора роста 1 (ИФР1) для лечения тяжелой инсулинорезистентности, в том числе и лепречаунизма.

В статье представлены клиническое описание пациента с синдромом Донохью и первый опыт применения непрерывной подкожной инфузии рекомбинантного ИФР1 при данном заболевании в России. По нашим наблюдениям, применение данной терапии оказалось эффективным. На фоне лечения в течение 15 мес отмечаются выраженная положительная динамика в отношении гликемического контроля, печеночной функции, а также регресс гипертрофической кардиомиопатии.

## Abstract (en)

Donohue syndrome (DS), also called Leprechaunism, is the most severe form of insulin resistance associated with biallelic mutations in INSR gene (OMIM: 147670). The approximate incidence of this syndrome is 1 per 1000000 births. Patients are present with typical clinical features such as intrauterine growth retardation, facial dysmorphism, severe metabolic disturbances, hepatomegaly and hypertrophic cardiomyopathy. Most DS patients die within the first two years of life due to respiratory infections, severe hypoglycemia or progressive cardiomyopathy. Treatment options are limited and no specific therapy exist for DS. Given the similarities between insulin and insulin-like growth factor 1 (IGF-1) receptors, recombinant human IGF-1 (rhIGF-1) has been used to treat severe insulin resistance including DS.

We report the case of a male patient with genetically confirmed Donohue syndrome, successfully treated with continuous subcutaneous IGF1 infusion via insulin pump. We observed improvement of glycemic control, liver function and cardiac hypertrophy regression following 15-month IGF1 therapy.

## АКТУАЛЬНОСТЬ

Синдром Донохью (лепречаунизм, OMIM *246200) — это наиболее тяжелая форма врожденной инсулинорезистентности, вызванная биаллельными мутациями в гене рецептора инсулина (INSR OMIM: 147670). Данное заболевание впервые было описано В.Л. Донохью и И. Учида в 1948 и 1954 гг. [1][2]. Клинические характеристики включают в себя выраженную задержку внутриутробного развития, дизморфизм («эльфоподобные» черты лица, липоатрофию, acanthosis nigricans, гипертрихоз), метаболические нарушения (постпрандиальную гипергликемию и гипогликемию натощак), гепатомегалию и гипертрофию миокарда [3]. Распространенность данного заболевания составляет порядка 1:1 000 000 живых новорожденных [4].

Большинство детей с диагнозом синдрома Донохью умирают в течение первых двух лет жизни, в основном в результате интеркуррентных инфекций верхних дыхательных путей, эпизодов гипогликемий или кардиомиопатии [5].

В настоящее время не существует специфической терапии при данном синдроме. В мировой литературе есть ряд описаний применения рекомбинантного инсулиноподобного фактора роста 1 (рИФР1) для лечения тяжелой инсулинорезистентности, вызванной мутациями в гене рецептора инсулина [3–5].

В данной статье мы представляем клиническое описание пациента с синдромом Донохью и первый опыт применения непрерывной подкожной инфузии рИФР-1 в России.

## ОПИСАНИЕ СЛУЧАЯ

Мальчик от неродственных здоровых родителей, шестой физиологической беременности, своевременных родов. Из наследственного анамнеза известно, что два сибса (мальчик и девочка) умерли в возрасте 4,5 и 3,5 мес. В обоих случаях отмечались прогрессирующая гипертрофическая кардиомиопатия, гепатомегалия и нарушения углеводного обмена. Генетическое обследование детям не проводилось.

Наш пациент родился с низкими весо-ростовыми показателями: масса тела 1788 г (SDS –3,09), длина тела 43 см (SDS –2,7), оценка по шкале Апгар 8/8 баллов. При осмотре обращали на себя внимание отсутствие подкожно-жирового слоя, смуглые и сухие кожные покровы, выраженный гипертрихоз, множественные стигмы диcэмбриогенеза.

В связи с явлениями задержки внутриутробного развития, отягощенной наследственностью и особенностями фенотипа ребенок был переведен в отделение патологии новорожденных, где при обследовании отмечалась гипергликемия максимальнодо 13 ммоль/л, находился на парентеральном питании. С 27 сут жизни регистрировалась стойкая гипергликемия, максимально до 24,7 ммоль/л. В гормональном профиле фиксировалось повышенное содержание уровня С-пептида (5291 пмоль/л при норме до 1730 пмоль/л). Проводилась инсулинотерапия без особой положительной динамики.

По результатам обследования в возрасте 1,5 мес у мальчика была выявлена гипертрофическая кардиомиопатия, инициирована терапия спиронолактоном и атенололом.

Пациент был заочно консультирован эндокринологом. На основании клинико-лабораторной картины был заподозрен синдром Донохью, который позже подтвержден молекулярно-генетически. Методом массового параллельного секвенирования на приборе Jon S5 проведен анализ 56 ядерных генов, ассоциированных с развитием врожденных нарушений обмена углеводов. В 4 экзоне гена INSR выявлен вариант: нуклеотидная замена NM 000208.3: chr19-7174702-A-G, c.1015T>C (p.Cys339Arg) вгомозиготном состоянии.

В возрасте 2 мес масса тела 2,93 кг (SDS -5,12), рост 48 см (SDS роста -5,08), при осмотре обращали на себя внимание типичные стигмы дисэмбриогенеза: микрогнатия, гипертрофия сосков, «лягушачий живот», преобладание мозгового отдела черепа над лицевым, отсутствие подкожно-жировой клетчатки, выраженный тотальный акантоз, гипертрихоз (рис. 1), задержка психомоторного развития, диффузная мышечная гипотония. Также отмечалась одышка до 50–60 дыхательных движений в минуту и тахикардия до 170 в минуту.

**Figure:**
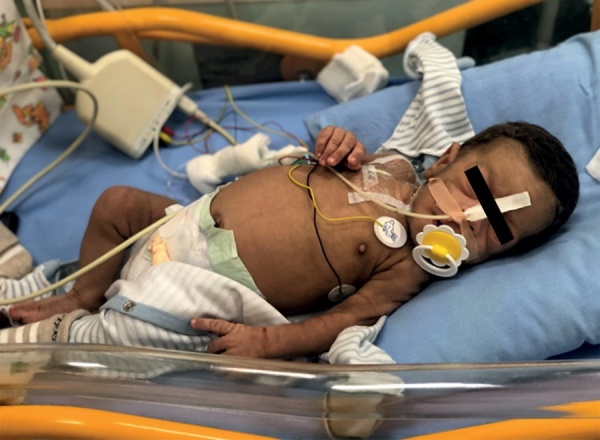
Рисунок 1. Пациент Н., 2 мес.Figure 1. Patient N., 2 months old.

Ребенок находился на дробном кормлении адаптированной детской смесью через соску в объеме 250 мл/кг/сут. На этом фоне регистрировались резкие перепады уровня сахара крови (от 1,4 через 1,5–2 ч после еды до 13 ммоль/л через 30–60 мин после еды; рис. 2). Клинических проявлений гипер/гипогликемии не отмечалось.

**Figure:**
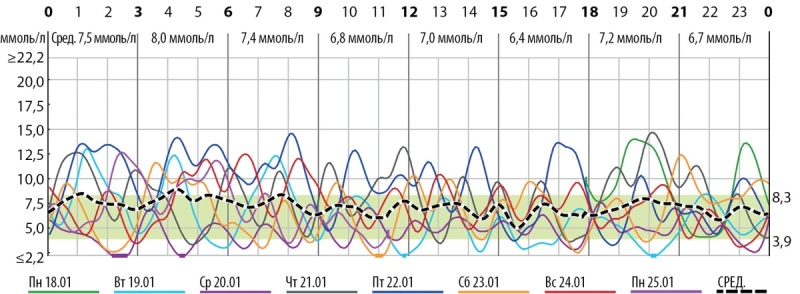
Рисунок 2. Данные непрерывного мониторирования гликемии на фоне дробного режима кормления: 60–70 г каждые 2,5–3 ч, 40 г каждые 1,5 часа.Figure 2. Data from continuous monitoring of glycemia against the background of a fractional feeding regimen: 60-70 g every 2.5-3 hours, 40 g every 1.5 hours.

При проведении гормонального исследования неоднократно фиксировались неопределяемо высокие уровни инсулина (>1000 мкЕ/мл) и С-пептида (>40 нг/мл), а также низкий уровень ИФР1 (9,37 нг/мл). Кроме того, были выявлены гипотироксинемия, явления цитолиза, гипокоагуляция (табл. 1). В возрасте 2,5 мес ребенок перенес массивное желудочно-кишечное кровотечение, потребовавшее гемотрансфузии.

**Table:** Таблица 1. Динамика результатов обследования на фоне проводимой терапии рекомбинантным ИФР-1Table 1. Dynamics of examination results against the background of therapy with recombinant IGF-1 Примечания: ИФР-1 — инсулиноподобный фактор роста -1; АЛТ — аланинаминотрансфераза; АСТ — аспартатаминотрансфераза; ТТГ — тиреотропный гормон; Т4св. — свободный тироксин; ТМЖПд — толщина межжелудочковой перегородки в диастолу; ТЗСЛЖд — толщина задней стенки левого желудочка в диастолу; УЗИ — ультразвуковое исследование; Эхо-КГ — эхокардиография; НГ-зонд — назогастральный зонд.

Возраст	2 мес	6 мес	10 мес	18 мес
Гликированный гемоглобин, % (4–6)	5,0	5,6	5,2	5,3
Инсулин, мкЕ/мл (2,6–24,9)	1000>	1000>	1000>	95,39
С-пептид, нг/мл (1,1–4,4)	40>	15,1	13,6	7,75
ИФР-1, нг/мл (11–233)	8,494	97,62	70,06	99,09
АЛТ, Ед/л (13–45)	73	123	70	124,7
АСТ, Ед/л (9–80)	89	176	94	86,9
ТТГ, мМЕ/л (0,98–5,63)	0,839	4,1	1,23	0,675
Т4св., пмоль/л (11,4–19,5)	7,74	9,03	12,75	14,17
Протромбиновое время, с (9,4–12,5)	20,8	17,5	14,3	15,1
Средняя упругость ткани печени, кПа	5,1 (SD±0,6)	5,7 (SD±0,3).	5,4 (SD±0,8)	5,7 (SD±0,8)
Размеры печени по УЗИ, см	6,0×3,2	8,0×3,1	8,0×3,6	8,1×3,9
Градиент давления в левом желудочке по данным Эхо-КГ, мм рт. ст.	В покое — 18.В беспокойном состоянии — 48	В покое — 44.В беспокойном состоянии — 66	4–6	5
ТМЖПд, мм (2–6)	12–13	13–14	11	6,4
ТЗСЛЖд, мм (3–6)	8,7	10	8,5	6,8
УЗИ почек	Без патологии	Паренхима: пирамидки с гиперэхогенными зонами, без акустической тени. Эхогенность коркового слоя выше нормы	Паренхима: пирамидки с гиперэхогенными зонами, без акустической тени. Эхогенность коркового слоя выше нормы	Паренхима: пирамидки с гиперэхогенными зонами, без акустической тени.Эхогенность коркового слоя выше нормы
Терапия	Не получал	Мекасермин 200 мкг/кг/сут + непрерывное кормление через НГ-зонд + дробное кормление перорально	Мекасермин 300 мкг/кг/сут + непрерывное кормление через НГ-зонд + дробное кормление перорально	Мекасермин 300 мкг/кг/сут + непрерывное кормление через НГ-зонд + дробное кормление перорально

В отделении было начато непрерывное зондовое кормление в стартовом объеме 80 мл/кг/сут с последующим наращиванием объема до 140 мл/кг/сут, на фоне которого отмечалась нормализация гликемии в течение дня (рис. 3).

**Figure:**
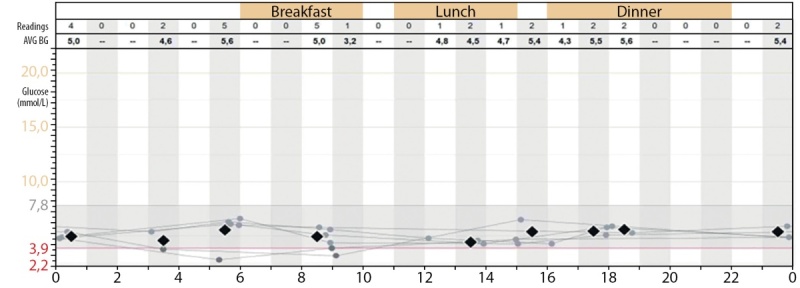
Рисунок 3. Непрерывное мониторирование гликемии на фоне непрерывного кормления через назогастральный зонд + дробное кормление перорально: 30–40 г каждые 2 ч; через назогастральный зонд со скоростью 20 мл/ч.Figure 3. Continuous monitoring of glycemia against the background of continuous feeding through a nasogastric tube + fractional feeding orally: 30-40 g every 2 hours; through a nasogastric tube at a rate of 20 ml / h.

В 3,5 мес было принято решение об инициации терапии рИФР1 — мекасермином (Инкрелексом) в стартовой дозе 100 мкг/кг/сут с постепенным увеличением до 200 мкг/кг/сут п/к каждые 8 ч. На фоне проводимой терапии отмечались рецидивы гипогликемий, несмотря на непрерывное кормление, что потребовало коррекции дозы (снижения до 150 мкг/кг/сут). Сохранялись выраженные отклонения в биохимическом анализе крови и по данным коагулограммы.

При повторном обследовании в 6 мес отмечалась прибавка массы тела (на момент обследования 4,49 кг, SDS веса -5,33), рост на момент поступления 57,5 см (-4,73 SDS). При осмотре обращали на себя внимание увеличение толщины подкожно-жировой клетчатки, уменьшение акантоза, улучшение моторных навыков. При проведении инструментального обследования была выявлена отрицательная динамика по данным эхокардиографии (нарастание градиента давления в левом желудочке до66 мм рт. ст.), по результатам ультразвукового исследования печени и почек отмечались гепатомегалия и диффузные изменения паренхимы почек. По данным лабораторного обследования отмечалось нарастание уровня трансаминаз, сохранялись гипотироксинемия и гипокоагуляция (табл. 1).

Был изменен режим введения препарата с дробного подкожного на непрерывный подкожный через инсулиновую помпу, что позволило нарастить дозу до 300 мкг/кг/сут, на фоне чего гипогликемии не фиксировались.

В возрасте 10 мес при очередном обследовании впервые зафиксирована положительная динамика по данным эхокардиографии (табл. 1).

На момент написания статьи пациенту 1 год 6 мес. Находится на прежней терапии, на фоне чего отмечается стойкая эугликемия (рис. 4). При осмотре явная положительная динамика в виде прибавки массы тела (7,5 кг; SDS веса -4,12) (рис. 5), удовлетворительной скорости роста (Δ SDS роста составила 0,8) (рис. 6), уменьшения акантоза, увеличения толщины подкожно-жировой клетчатки (рис. 7), улучшения неврологических показателей. При лабораторном обследовании: значимое улучшение биохимических показателей, нормализация тиреоидного профиля и коагулограммы (табл. 1). При проведении эхокардиографии в динамике отмечается улучшение результатов, в настоящее время обструкция в левом желудочке не выявляется.

**Figure:**
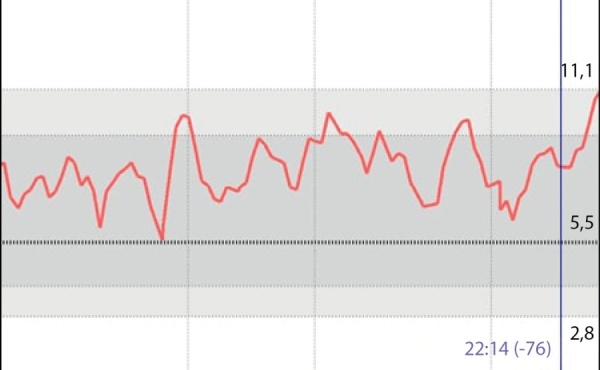
Рисунок 4. Flash-мониторинг на фоне терапии мекасермином + непрерывное питание через назогастральный зонд.Figure 4. Flash monitoring during therapy with mecasermin + continuous feeding through a nasogastric tube.

**Figure:**
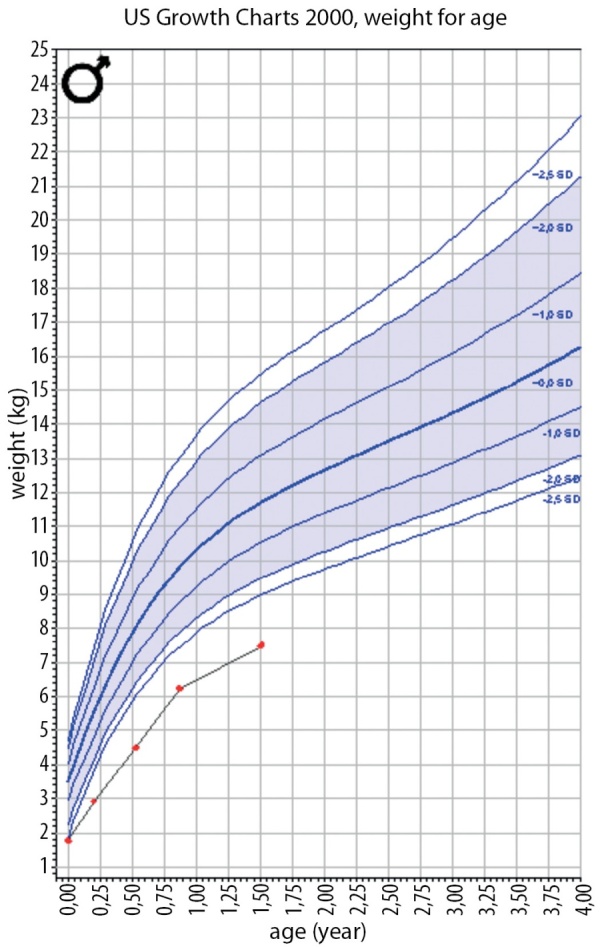
Рисунок 5. График SDS массы тела пациента Н.Figure 5. SDS plot of patient N's body weight.

**Figure:**
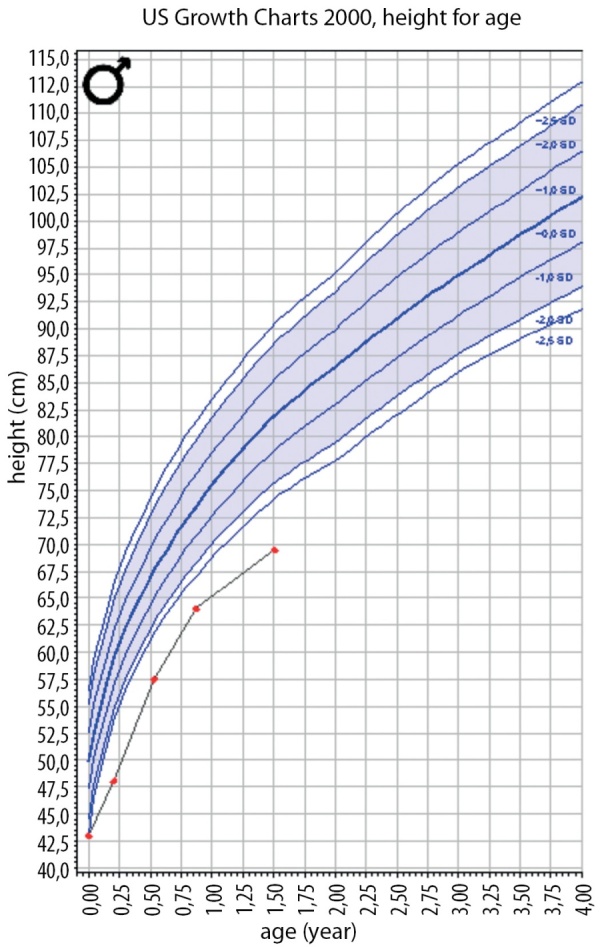
Рисунок 6. График SDS роста пациента Н.Figure 6. SDS plot of patient H growth

**Figure:**
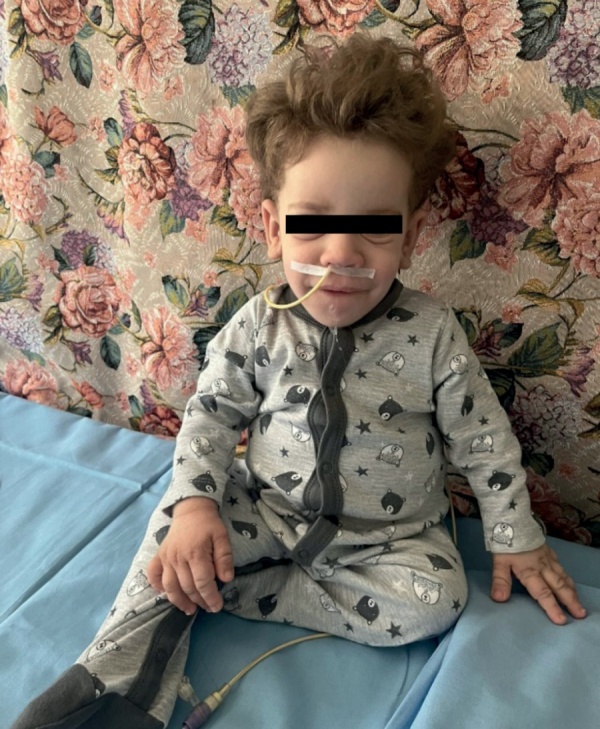
Рисунок 7. Пациент Н., 18 мес.Figure 7. Patient N., 18 months old

## ОБСУЖДЕНИЕ

Инсулиновый рецептор (ИР) — это тирозинкиназный трансмембранный рецептор, представляющий собой гетеротетрамер, состоящий из 2 α-субъединиц и 2 β-субъединиц. ИР экспрессируется на мембране инсулинзависимых клеток, широко представленных практически во всех органах, в особенности в печени, мышечной и жировой ткани, и активируется посредством связывания с инсулином, а также инсулиноподобными факторами роста I и II. В активированном состоянии ИР запускает каскад внутриклеточных реакций, ответственных в том числе за транспорт глюкозы, синтез гликогена, аутофагию, клеточную пролиферацию, апоптоз и экспрессию других генов. ИР кодируется единственным геном INSR, который состоит из 22 экзонов. Мутации в гене INSR могут приводить к различным вариантам инсулинорезистентности, наиболее тяжелым из которых считается синдром Донохью. Данное заболевание ассоциировано с гомозиготными или компаундными гетерозиготными мутациями, приводящими к полной потере способности рецептора связываться с инсулином или же его дефектному формированию, препятствующему адекватной экспрессии на мембранах клеток. На сегодняшний день описано уже более 150 различных мутацийв INSR, приведших к развитию синдрома Донохью. Большая часть этих мутаций приходится на α-субъединицу, структурирующую экстрацеллюлярную часть рецептора, ответственную за аффинность рецептора с инсулином, в то время как β-субъединица формирует внутриклеточную область рецептора и играет ключевую роль в процессах аутофосфорилирования. Биаллельные мутации в регионе, кодирующем β-субъединицу, чаще сопряжены с более мягким фенотипом, синдромом Рабсона–Менденхолла [6].

Мутация, выявленная у нашего пациента, ранее не описана в литературе и отсутствует в международной базе HGMD. Данный вариант нуклеотидной последовательности затрагивает L2 домен (богатый лейцином повторяющийся домен 2) α-субъединицы ИР. В литературе имеются данные функциональных исследований L2 мутантного рецептора, демонстрирующие значимое снижение экспрессии зрелых рецепторов на клеточной мембране, а также полную утрату их лигандной способности к инсулину [7].

Клиническая диагностика синдрома Донохью, как правило, не вызывает особых сложностей ввиду яркого фенотипа и характерных флюктуаций гликемии (высокая ранняя постпрандиальная гипергликемия с последующей выраженной гипогликемией через 1–2 ч после еды). Отсрочка в постановке диагноза может быть обусловлена крайней редкостью данного синдрома.

У нашего пациента отмечались типичные для заболевания фенотипические и лабораторные изменения, а также отягощенный наследственный анамнез (два сибса умерли в младенческом возрасте от обструктивной гипертрофической кардиомиопатии и имели схожие фенотипы).

Клинические проявления у описанного нами ребенка являются отражением дисфункции всех метаболических функций посттрансляционного сигналинга инсулина. Так, помимо выраженной нестабильности гликемического профиля, ассоциированной с неспособностью организма к синтезу гликогена, в структуре заболевания доминировали проявления полиорганной дисфункции с формированием обструктивной гипертрофической кардиомиопатии, тубулярной нефропатии ибелково-синтетической недостаточности печени (табл. 1). Кроме того, отмечалась грубая задержка психомоторного развития, которая была ассоциирована как с мышечной дистрофией, так и, вероятно, с перенесенными в первые дни-недели жизни гипогликемиями.

Поддержание эугликемии у пациентов с синдромом Донохью представляет большие сложности. Так, для предотвращения гипогликемии и сглаживания постпрандиальных гипергликемий в нашем случае потребовалось проведение непрерывного капельного кормления в течение суток (рис. 4). Потребность в данном режиме вскармливания сохраняется у описанного нами ребенка по настоящее время, несмотря на набор массы тела и формирование подкожно-жирового слоя.

Одним из наиболее грозных и зачастую фатальных проявлений заболевания является гипертрофическая кардиомиопатия (ГКМП), также обнаруженная у нашего пациента. По данным литературы, ГКМП у детей с синдромом Донохью выявляется в трети случаев и в 85% приводит к острым нарушениям ритма и летальному исходу [5]. Патогенез формирования ГКМП при данном заболевании на сегодняшний день остается до конца неясным. Считается, что высокие концентрации инсулина в крови способны связываться с рецепторами к ИФР1, а также гибридными инсулин-ИФР1-рецепторами (ИФР1-Р), экспрессированными в сердечной мышце, что приводит к избыточной пролиферации кардиомиоцитов. Данная теория подтверждается клиническими наблюдениями диспропорциального разрастания хрящевой ткани, богатой ИФР1-Р, у пациентов с врожденными формами инсулинорезистентности. В то же время исследования последних лет демонстрируют критическую роль сигнального пути инсулина в миокарде и клетках сосудов [8][9]. Отсутствие активации PKB/Akt/TSC2/mTOR каскадного пути субстратами ИР приводит к снижению захвата глюкозы, оксидативному стрессу, митохондиальной дисфункции и уменьшению высвобождения оксида азота в клетках эндотелия, что влечет за собой дистрофические изменения в сердечной мышце и снижение способности к вазодилятации [10][11].

На сегодняшний день патогенетического лечения для синдрома Донохью не существует. В литературе есть единичные упоминания об относительно успешном применении метформина и препаратов лептина при тяжелых вариантах наследственной инсулинорезистентности [12][13]. Учитывая структурную схожесть инсулина, проинсулина, ИФР1 и ИФР2, а также гомологичность рецепторов к инсулину и к ИФР1, рИФР1 был предложен как возможная терапевтическая опция для пациентов с наследственной инсулинорезистентностью, позволяющая в обход сломанного ИР активировать его сигнальные пути [14].

ИФР1-Р представляет собой трансмембранный тирозинкиназный рецептор, схожий по структуре с ИР. Он имеет высокую лигандную аффинность с ИФР1 и ИФР2 и слабо связывается с инсулином. ИФР1-Р регулирует рост, дифференцировку и апоптоз клеток. В активном состоянии аутофосфорилирование его тирозинкиназного домена приводит к активации P13K-PKB/Akt-пути, играющего ключевую роль в метаболизме глюкозы [15][16].

У здорового человека инфузия рИФР1 подавляет выработку глюкозы в печени, стимулирует периферический захват глюкозы в мышцах и, несмотря на значительное снижение уровней циркулирующего инсулина, вызывает гипогликемию [17].

У пациентов с диабетом 1 или 2 типа рИФР1 снижает уровень глюкозы в крови аналогично экзогенному инсулину; однако побочные эффекты (отек лица и рук, артралгии, миалгии и тахикардия) ограничивали его использование в ситуациях, когда введение инсулина было эффективным [18][19].

Первые сообщения об использовании рИФР1 у пациентов с синдромом инсулинорезистентности были опубликованы в 1990-х годах. При однократном внутривенном введении рекомбинантного ИФР1 в дозе 100 мкг/кг/день отмечалось снижение концентраций глюкозы, инсулина и С-пептида в крови [20][21]. За этими первоначальными испытаниями последовали исследования с применением подкожного введения рИФР1 [22–28]. H. Kuzuya и соавт. впервые описали терапию рИФР1 в течение нескольких месяцев у пациентов с синдромом инсулинорезистентности. В результате лечения у пациентов отмечалось значимое снижение уровня HbA1с [29]. M. de Kerdanet и соавт. описывают опыт применения терапии рИФР1 у пациента с синдромом Донохью в течение 10 лет с положительным эффектом. В данном исследовании указаны небольшие дозы рИФР1, при лечении которыми достигался адекватный уровень рИФР1 и регистрировалась положительная динамика в виде нормализации гликемии, улучшения функции печени и ускорения темпов роста и набора массы тела [30].

Описанный нами случай демонстрирует эффективность проводимой терапии рИФР1 — в динамике у пациента отмечаются улучшение темпов роста, значимая прогрессия в наборе веса, выраженная положительная динамика метаболических показателей и функции печени. Кроме того, при последнем очередном обследовании нами было зарегистрировано уменьшение обструкции в сердце (табл. 1).

В публикациях последних лет авторы отдают предпочтение непрерывной подкожной инфузии ИФР1, что позволяет избежать перепадов концентрации препарата в крови и снижает риски гипогликемии [28].

В случае с нашим пациентом непрерывный режим введения ИФР1 очевидно имел преимущества, что подтверждается данными непрерывного мониторирования гликемии (рис. 4).

## ЗАКЛЮЧЕНИЕ

Несмотря на относительно длительный опыт применения рекомбинантного ИФР1 в лечении различных патологий, данные о его использовании у пациентов с синдромом Донохью лимитированы единичными публикациями. Большинство из них представляет собой либо клинические описания отдельных случаев, либо включает небольшие группы пациентов с синдромом инсулинорезистентности с выраженными различиями в клинических фенотипах (от синдрома Донохью и синдрома Рабсона–Менденхолла до инсулинорезистентности типа А) [22–29]. Результаты терапии существенно варьируют от публикации к публикации, что делает невозможной адекватную оценку эффективности данного лечения. Кроме того, в ряде статей оговариваются потенциальные побочные эффекты терапии рИФР1, в частности риски опухолевого роста, развитие пролиферативной ретинопатии, а также усугубление течения ГКМП [31][32].

У наблюдаемого нами ребенка к настоящему моменту не отмечено указанных осложнений терапии. Более того, наоборот, мы видим регресс ГКМП в динамике. Однако малая на сегодняшний день продолжительность наблюдения не позволяет нам исключить рисков возникновения побочных реакций на терапию.

## ДОПОЛНИТЕЛЬНАЯ ИНФОРМАЦИЯ

Источники финансирования. Работа выполнена по инициативе авторов без привлечения финансирования.

Конфликт интересов. Авторы декларируют отсутствие явных и потенциальных конфликтов интересов, связанных с содержанием настоящей статьи.

Участие авторов. Иванникова Т.Е. — вклад в концепцию и дизайн исследования, получение и анализ данных, написание статьи; Меликян М.А., Колодкина А.А., Безлепкина О.Б., Мокрышева Н.Г. — анализ данных, интерпретация результатов, существенный вклад в дизайн исследования и интерпретацию результатов, редакция текста; Милованова Н.В. — интерпретация результатов, внесение в рукопись существенной правки с целью повышения научной ценности статьи. Все авторы одобрили финальную версию статьи перед публикацией, выразили согласие нести ответственность за все аспекты работы, подразумевающую надлежащее изучение и решение вопросов, связанных с точностью или добросовестностью любой части работы.

Согласие пациента. Законный представитель пациента добровольно подписал информированное согласие на публикацию персональной медицинской информации в обезличенной форме в журнале «Проблемы эндокринологии».
